# Autophagy Interplay with Apoptosis and Cell Cycle Regulation in the Growth Inhibiting Effect of Resveratrol in Glioma Cells

**DOI:** 10.1371/journal.pone.0020849

**Published:** 2011-06-13

**Authors:** Eduardo C. Filippi-Chiela, Emilly Schlee Villodre, Lauren L. Zamin, Guido Lenz

**Affiliations:** 1 Department of Biophysics, Universidade Federal do Rio Grande do Sul (UFRGS), Porto Alegre, RS, Brazil; 2 Universidade Federal da Fronteira Sul (UFFS), Cerro Largo, RS, Brazil; 3 Center of Biotechnology; Universidade Federal do Rio Grande do Sul (UFRGS), Porto Alegre, RS, Brazil; Istituto Nazionale per le Malattie Infettive, Italy

## Abstract

Prognosis of patients with glioblastoma (GBM) remains very poor, thus making the development of new drugs urgent. Resveratrol (Rsv) is a natural compound that has several beneficial effects such as neuroprotection and cytotoxicity for several GBM cell lines. Here we evaluated the mechanism of action of Rsv on human GBM cell lines, focusing on the role of autophagy and its crosstalk with apoptosis and cell cycle control. We further evaluated the role of autophagy and the effect of Rsv on GBM Cancer Stem Cells (gCSCs), involved in GBM resistance and recurrence. Glioma cells treated with Rsv was tested for autophagy, apoptosis, necrosis, cell cycle and phosphorylation or expression levels of key players of these processes. Rsv induced the formation of autophagosomes in three human GBM cell lines, accompanied by an upregulation of autophagy proteins Atg5, beclin-1 and LC3-II. Inhibition of Rsv-induced autophagy triggered apoptosis, with an increase in Bax and cleavage of caspase-3. While inhibition of apoptosis or autophagy alone did not revert Rsv-induced toxicity, inhibition of both processes blocked this toxicity. Rsv also induced a S-G2/M phase arrest, accompanied by an increase on levels of pCdc2(Y15), cyclin A, E and B, and pRb (S807/811) and a decrease of cyclin D1. Interestingly, this arrest was dependent on the induction of autophagy, since inhibition of Rsv-induced autophagy abolishes cell cycle arrest and returns the phosphorylation of Cdc2(Y15) and Rb(S807/811), and levels of cyclin A, and B to control levels. Finally, inhibition of autophagy or treatment with Rsv decreased the sphere formation and the percentage of CD133 and OCT4-positive cells, markers of gCSCs. In conclusion, the crosstalk among autophagy, cell cycle and apoptosis, together with the biology of gCSCs, has to be considered in tailoring pharmacological interventions aimed to reduce glioma growth using compounds with multiple targets such as Rsv.

## Introduction

Glioblastoma (GBM) are the most common primary brain tumors, with a worldwide annual incidence of around 7 cases per 100,000 individuals [1], [2]. More than 20,000 cases are diagnosed every year only in the USA and gliomas have a disproportionately high mortality rate of more than 70% of cases in two years after diagnosis (www.cancer.org; http://www.cbtrus.org) [2]. Among the primary brain tumors, GBM, classified as grade IV by the World Health Organization, is the most frequent and biologically aggressive type, corresponding to around 65% of cases [2], [3]. The high malignancy of GBM is due to their intense cell proliferation, diffuse infiltration, high resistance to apoptosis [1], [2] and robust angiogenesis, in which cells from the tumor form part of the endothelium possibly due to reprogramming [4], [5], [6]. GBM Cancer Stem Cells (gCSC) have received much attention in glioma biology and this type of cell is highly associated with high aggressiveness, being fundamental for the maintenance and recurrence of GBM [7]. It was recently shown that gCSCs participate in the formation of the tumor endothelium [8], increasing the invasiveness of the tumor [9] and leading to the resistance to radiotherapy [10], [11] through several mechanisms. The primary therapy for GBM consists in surgery followed by radio and chemotherapy with temozolomide (TMZ), which is in clinical use since 2005 [11], [12], [13], [14]. Despite this multimodal approach, the prognosis has only slightly improved [1], [2].

Among the potential alternatives that have emerged for treating GBM are some natural products which present high antitumoral efficiency without some of the harmful side effects of conventional chemotherapies. Resveratrol (Rsv) (3,4,5-trihydroxy-*trans*-stilbene) is a polyphenolic phytoalexin widely present in plants and enriched in red wine, peanuts and other sources [15], [16]. This compound exerts beneficial functions in normal cells both *in vitro* and *in vivo*, by inducing mitochondrial biogenesis [17] and neuroprotection in adverse conditions, such as oxygen-glucose deprivation [18] and traumatic brain injury [19]. On the other hand, it is cytotoxic for the majority of malignant cells, blocking the three major stages of carcinogenesis (*i.e.* initiation, promotion and progression) [19] in several types of cancer cells and models, like breast [20], colon [21], melanoma [22], uterine [23], lung [24] and leukemia cells [25]. Rsv exerts its toxicity through modulation of several pathways and induction of different mechanisms of cell death and growth inhibition [26], [27]. It induces apoptosis in colon cancer cells [28], necrosis in prostate carcinoma cells [29], growth arrest in myeloma cells [30] and autophagocytosis in ovarian cancer cells [31]. In gliomas, Rsv induces signs of necrosis, apoptosis and senescence in C6 (rat) cells [32], apoptosis in U251 and U87 (human) cells in high doses [33], [34] and autophagy in U251 cells [34]. In C6, U138 (human) and GL261 (mouse) glioma cells lines, we have previously shown that Rsv inhibits cells growth, through mechanisms that involve, but are not restricted to, apoptosis and senescence [32].

Growth inhibition and induction of cell death are among the major objectives of anti-cancer therapies. Some types of cancers frequently develop resistance to apoptotic cell death, among which we highlight primary gliomas. Two important pathways mediate part of this resistance in these tumors: PTEN/Akt/PI3K pathway, which is over activated in GBM cells through loss of PTEN, over expression of EGFR (a typical alteration of primary gliomas) and/or increase of PI3k/Akt activity due to mutations in its regulators; and NF-κB pathway, which is constitutively activated in a large proportion of GBMs and is increased by cells in response to cytotoxic drugs, favoring cell survival by inducing the expression of anti-apoptotic genes. Thus, inhibition of these pathways may be a way to decrease GBM intrinsic- and drug-induced resistance, sensitizing GBM cells to apoptotic cell death. On the other hand, induction of other non apoptotic mechanisms of cell death are central for the elimination of apoptosis-resistant GBM cells [35]. Thus, the development of drugs that induce multiple mechanisms of cell death like senescence, mitotic catastrophe, paraptosis, autoschizis and specially autophagy and autophagic cell death are fundamental to overcome this resistance [36], [37], [38], [39]. Autophagy is a genetically programmed, evolutionarily conserved process coordinated by a family of genes, called Atg, that lead to the degradation of organelles and proteins. It involves the formation of double-membrane vesicles, containing cellular components, that merge to lysosomes, forming the autophagolysosome, where the components are degraded and the products generated are reused by the cell [40], [41]. In this view, autophagy acts as a prosurvival mechanism, mainly in adverse conditions such as nutrient and oxygen deprivation. However, this process has a clear self-limiting character and may lead to cell death with autophagic features (or programmed cell death type II) when at high levels or duration [42].

In cancer, it has been shown that autophagy may be an important anti-cancer mechanism *in vivo* since the expression level of beclin-1, a fundamental gene for autophagy, is inversely correlated with the malignancy of brain tumors [43] and is directly correlated with survival [44]. Moreover, autophagy is induced by efficient physical and chemical anti-cancer treatments in gliomas, like TMZ, rapamycin, γ radiation, oncolytic adenoviruses and others [31], [45], [46], [47], [48], [49]. Moreover, it was shown that GBM cells are more sensitive to agents that induce autophagy than apoptosis, like TMZ [46], [50], and autophagic structures were found in gliomas *in vivo* after treatments [51]. Increasingly, the understanding of the complexity of the relationship between apoptotic cell death and autophagy (and other mechanisms of cell death and growth inhibition) in cancer is required for the understanding on how to tip the balance from tumor survival to death [52].

Here we evaluated the actions of Rsv in glioma cells, focusing on the role of autophagy and their interaction with cell cycle regulation, apoptosis and the biology of gCSCs. We showed that Rsv induced autophagy and S-G2/M cell cycle arrest, but not necrosis or apoptotic cell death, in human GBM cells. Inhibition of basal autophagy decrease the stemness of GBM cells, while inhibition of Rsv-induced autophagy in U87 cells caused apoptotic cell death and, more interestingly, inhibited cell cycle arrest induced by Rsv, suggesting that, despite not being directly involved in the inhibition of cell growth by Rsv, autophagy plays an indirect, but fundamental, role in mediating the effects of Rsv in GBMs.

## Results

### Rsv induces autophagosome formation in U87 glioma cells

**Figure 1:**
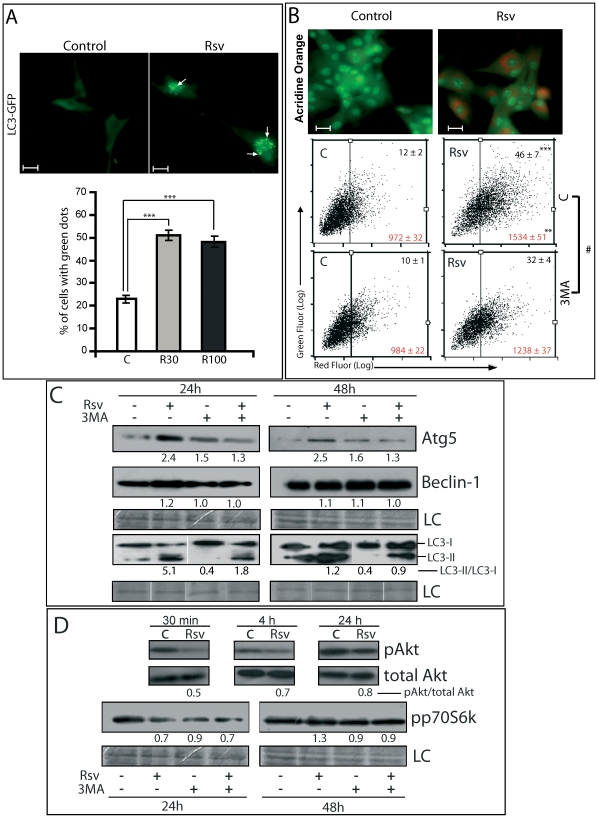
Rsv induces autophagy in glioma cells. **(A)** U87 cells were transfected with pEGFP-LC3 and 48 h later treated with Rsv 30 µM or 100 µM for 48 h. **Top** - Representative images of cells with cytosolic green dots, representing LC3-GFP marked autophagosomes (white arrows), in cells treated with Rsv 30 µM; scale bar: 10 µm. **Lower Pannel:** Percentage of cells treated with Rsv 30 or 100 µM for 48 h which presented more than five defined cytosolic green dots. *** p<0.001; **(B)** U87 cells were pre-incubated with buffer **(top and middle)** or 3MA (2 mM) **(bottom)** for 1 h, treated with Rsv 30 µM for 48 h, followed by acridine orange (AO) staining and flow cytometry. **Top** - Representative images of AO stained cells treated with Rsv 30 µM for 48 h – acidic vacuolar organelles (AVO) stain red; scale bar: 20 µm. **Middle and bottom** - percentage of cells with positive red fluorescence as analyzed by flow cytometry. Numbers in the quadrants refer to average of events (black) or X-mean of AO red fluorescence intensity (red) ± SEM of three independent experiments; **p<0.01, ***p<0.001 in relation to control as indicated; # p<0.01, 3MA versus C for Rsv treated cells; **(C and D)** U87 cells were treated as in B and western blots for the indicated proteins were performed at the indicated time. Numbers indicate the band intensity in relation to control. LC: Loading control – coomassie stained PVDF membrane.

We previously showed that Rsv inhibited the growth of glioma cells through processes that included senescence and apoptosis [32]. Here we show that treatment of U87 glioma cell line with Rsv at the relatively low concentration of 30 µM increased the percentage of cells with LC3-GFP cytosolic dots representing autophagosomes (Fig. 1A). This effect was not further increased with higher concentrations of Rsv, reaching a plateau of around 50% LC3-GFP positive cells, as previously observed in other cells [53]. Autophagosomes were also observed through AO staining, which significantly increased after Rsv treatment (Fig. 1B). 3MA, an inhibitor of the enzyme phosphatidylinositol 3-kinase class III (PI3k class III), essential for the autophagic process [54], reduced the number of cells containing LC3-GFP marked autophagosomes from 19% to 9% under basal conditions and from 55% to 24% when treated with Rsv 30 µM for 48 h. Similarly, the proportion of cells with red staining and the intensity of red staining with AO increased with Rsv and was partially reverted with 3MA, indicating that inhibition of PI3k class III not only reduced the number of cells undergoing autophagy, but also reduced the number of mature autophagosomes formed per cell (Fig. 1B - bottom).

To molecularly confirm the induction of autophagy, we measured the expression of autophagy-related proteins. Rsv induced an increase in Atg5 and the lipidated form of LC3 (LC3-II) at 24 and 48 h, further evidence for the induction of autophagy (Fig. 1C). This may be mediated by the reduction in the activity of Akt or p70S6K, as indicated by the reduction in its phosphorylation at early time points of treatment (Fig. 1D). 3MA significantly blocked the effects of Rsv on Atg5 and LC3-II but not on p70S6K phosphorylation (Fig. 1C, D).

### Rsv-induced autophagy is not directly responsible for its cytotoxicity

**Figure 2:**
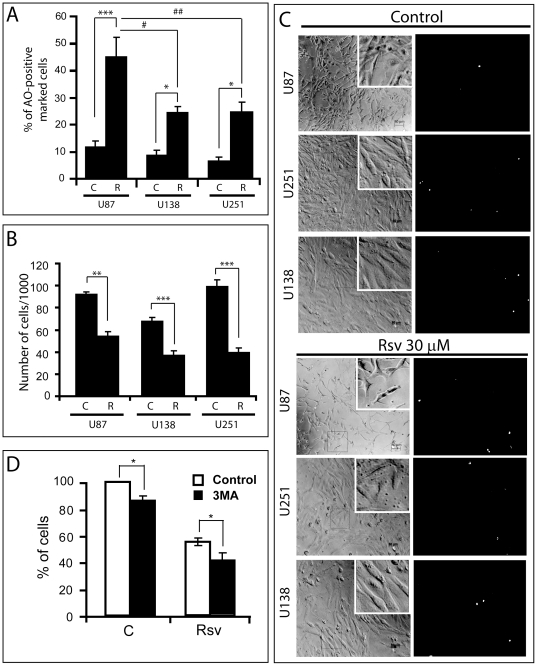
Rsv-induced autophagy acts as a prosurvival mechanism in glioma cells. U87, U138 and U251 glioma cells were treated with Rsv 30 µM for 48 h, followed by **(A)** AO red fluorescence quantification flow cytometry, **(B)** cell counting and **(C)** morphological appearance (left) and assessment of plasma membrane integrity with Propidium Iodide (PI) (right) (white dots represents PI-positive cells). In **(D)**, U87 cells were pre-incubated with 3MA (2 mM) for 1 h, followed by treatment with Rsv 30 µM for 48 h and cell counting; * p<0.05, ** p<0.01, *** p<0.001 in relation to control as indicated; ^#^ p<0.05, ^##^ p<0.01 in relation to U87 cells treated with Rsv 30 µM.

A direct comparison of the level of autophagosome formation in three glioma cell lines showed that Rsv induced higher autophagosome levels in U87 cells when compared to p53 negative cell lines U251 and U138 (Fig. 2A). Notwithstanding, Rsv reduced the cell number of all three cell lines, even inducing a larger effect in U138 and U251 when compared to U87 (Fig. 2B). Treatment with 30 µM of Rsv for 48 h, however, did not significantly change cell morphology or induced necrosis in three GBM cell lines tested (Fig. 2C). Inhibition of Rsv-induced autophagy with 3MA did not block cell number reduction caused by Rsv in U87 cells, even slightly potentiating the effect of Rsv (Fig. 2D), suggesting that Rsv-induced autophagy acts as a slight cytoprotective mechanism rather than playing a direct role in the cytostatic/cytotoxic mechanism of Rsv in U87 cells.

### Inhibition of Rsv-induced autophagy increases apoptosis in U87 cells

**Figure 3:**
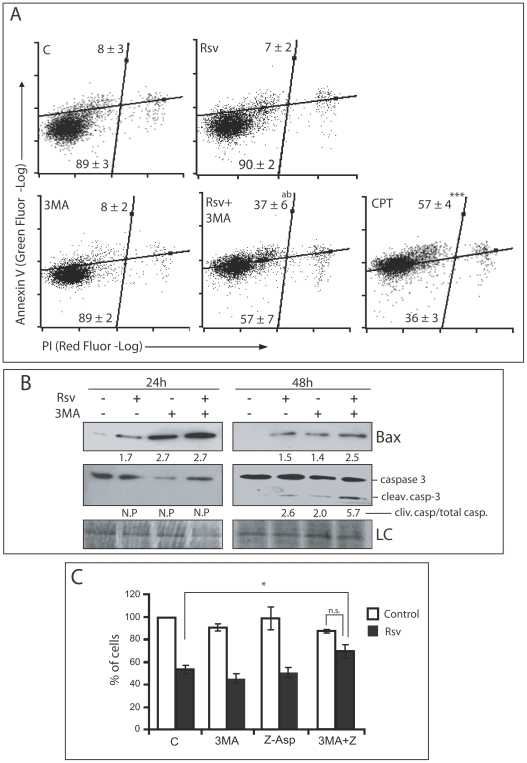
Inhibition of Rsv-induced autophagy causes apoptosis. **(A)** U87 cells were pre-incubated with 3MA (2 mM) for 1 h before treatment with Rsv 30 µM for 48 h, followed by annexin V-FLUOS/PI cell staining evaluated by flow cytometry. Numbers in quadrants represents the respective percentage of cells ± SEM of three independent experiments. ^a^ p>0.01 and ^b^ p>0.01 in relation to Rsv and 3MA, respectively; *** p<0.001 in relation to control. **(B)** U87 cells were treated as in A and lysed 24 or 48 h later and western blots for the indicated proteins were performed. Numbers indicate the band intensity in relation to control. LC: Loading control. **(C)** U87 cells were pre-incubated with 3MA (2 mM) and/or zAsp (100 µM) for 1 h followed by treatment with Rsv (30 µM) for 48 h and number of cells was determined in a hemocytometer. Data are given as media ± SEM and are expressed as percentage of control, considered 100%; * p>0.05; n.s. not significant.

As cited above, Rsv did not significantly change cell morphology or induce necrosis in GBM cells (Fig. 2C). Treatment with 30 µM of Rsv also did not increase phosphatidylserine (PS) externalization, evaluated by annexin V-staining (Fig. 3A), but induced a small increase in Bax expression and caspase cleavage (Fig. 3B). When Rsv-induced autophagy was inhibited with 3MA, a strong increase in the proportion of annexin V-positive cells was observed (Fig. 3A), together with a significant increase in Bax expression and caspase 3 cleavage (Fig. 3B). This was accompanied by phenotypic alterations indicating apoptosis, *i.e.* rounded morphology and loss of surface adhesion (Fig. S1). Inhibiting apoptosis by using a pan caspase inhibitor (zAsp) or inhibiting autophagy with 3MA alone did not block the cytotoxicity induced by Rsv. However, when both autophagy and apoptosis inhibitors were present, the Rsv-induced reduction in cell number was significantly inhibited (Fig. 3C).

### Rsv induces a S-G2/M cell cycle arrest in an autophagy-dependent way

**Figure 4:**
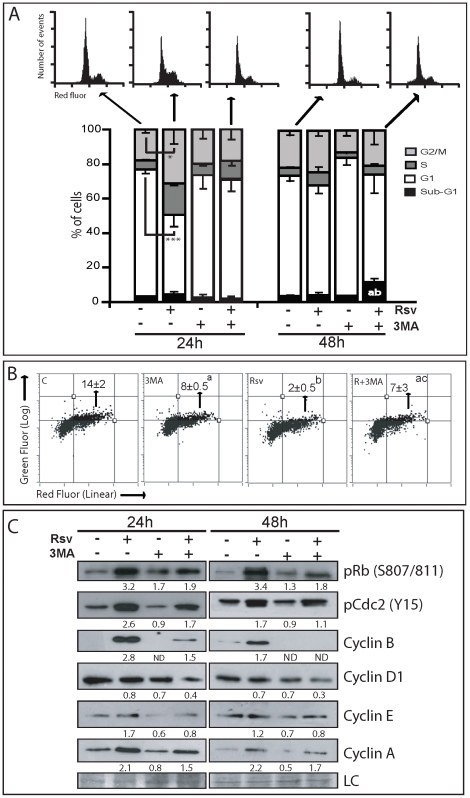
Autophagy mediates Rsv-induced S-G2/M cell cycle arrest in U87 cells. **(A)** U87 cells were pre-incubated with 3MA (2 mM) for 1 h, followed by treatment with Rsv (30 µM) for 24 h or 48 h and cell cycle analysis by flow cytometry; * p<0.05, *** p<0.001; ^a^ and ^b^ p<0.05 in relation to Rsv and control 24 h, respectively. **(B)** BrdU incorporation assay. U87 cells were treated as in A for 24 h. From 21 to 24 h after treatment, cells were incubated with 10 µM of BrDU. After that, cells were fixed, stained and analyzed by flow cytometry. Graph indicates the percentage of BrdU-positive cells± SEM; ^a^ p>0.001 and ^b^ p>0.01 in relation to control; ^c^p>0.05 in relation to Rsv alone; **(C)** Cells were treated as in A, followed by western blotting using the indicated antibodies. Numbers indicate the band intensity in relation to control. LC – loading control.

Because Rsv was shown to inhibit cell cycle progression in several cancer types (fully reviewed in [55]), we tested if it modulates cell cycle dynamics in GBM cells. Rsv induced a transient S-G2/M cell cycle arrest after 24 h of treatment, whereas at 48 h, the cell cycle distribution returned to control levels (Fig. 4A). Interestingly, inhibition of autophagy completely blocked the Rsv-induced cell cycle arrest (Fig. 4A). Evaluation of DNA synthesis through BrdU incorporation assay showed that Rsv significantly reduced DNA synthesis rate after 24 h, suggesting that cells remained with its DNA partially duplicated, but without further synthetizing DNA. Inhibition of autophagy partially reverted this block in DNA synthesis (Fig. 4B).

The arrest induced by Rsv was accompanied by an increase in the phosphorylation of CDC2 (Cdk1) on Tyr15, pRb and an increase in the expression (or stabilization) of cyclin A, B and E, but not cyclin D1 (Fig. 4C). Similarly to U87 cells, U138 cells presented an increase in S phase and U251 increased S and G2/M phases upon treatment with 30 µM Rsv for 48 h (Fig. S2), showing that modulation of cell cycle by this dose of Rsv in glioma cells was not cell line-specific and may explain, at least partially, the reduction in cell number after Rsv treatment.

In an attempt to find the signaling that coordinates the link between cell cycle and autophagy, we observed that inhibition of autophagy partially reverted the effects of Rsv on pCDC2(Y15), pRb, cyclin A, cyclin B and cyclin E. On the other hand, inhibition of Rsv-induced autophagy caused a decrease in cyclin D1 levels (Fig. 4C). It is important to point out that after 48 h, treatment of Rsv in the presence of 3MA led to a slight increase in the sub-G1 population (Fig. 4A) in U87 cells, in accordance with observations described above that inhibition of Rsv-induced autophagy triggered apoptosis in these cells.

### Blocking of basal autophagy reduces stemness of gCSCs

**Figure 5:**
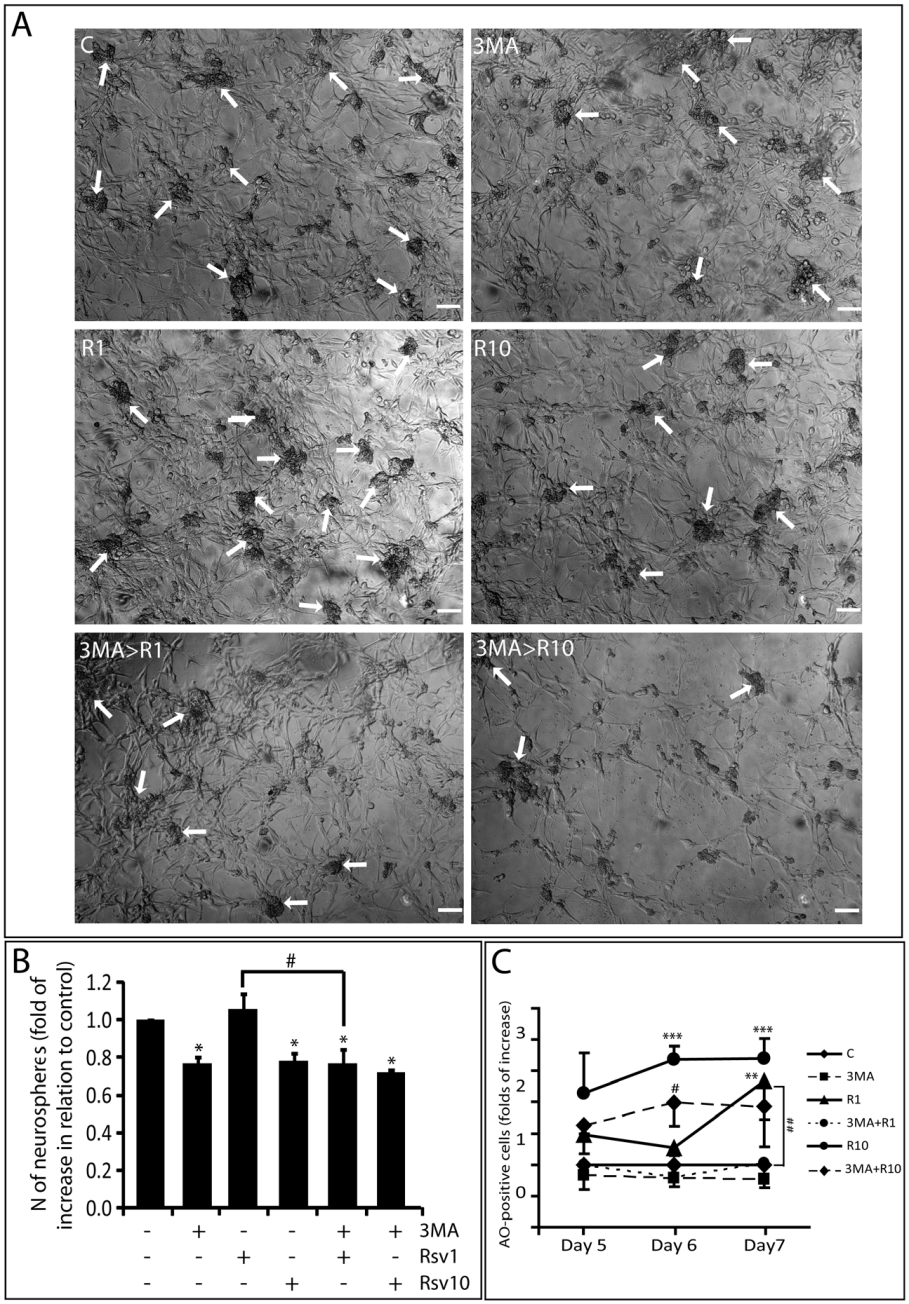
Rsv or autophagy inhibition reduces sphere formation in gliomas. Cells were pre-incubated with 2 mM of 3MA or buffer for 1 h, followed by treatment with Rsv as indicated; on day 4 the medium was changed and new drugs were added; on day 7, number of spheres was counted; **(A)** representative images of spheres formed after 7 days of treatment; white arrows point to spheres; scale bar: 30 µm; **(B)** number of spheres after 7 days of treatment; **(C)** Levels of autophagy from 4 to 7 days after treatment measured with AO; ** and *** p>0.001 and p>0.001 in relation to control for the same time point; # and ## p>0.05 and p>0.01 in relation to Rsv alone, for the same dose.

**Figure 6:**
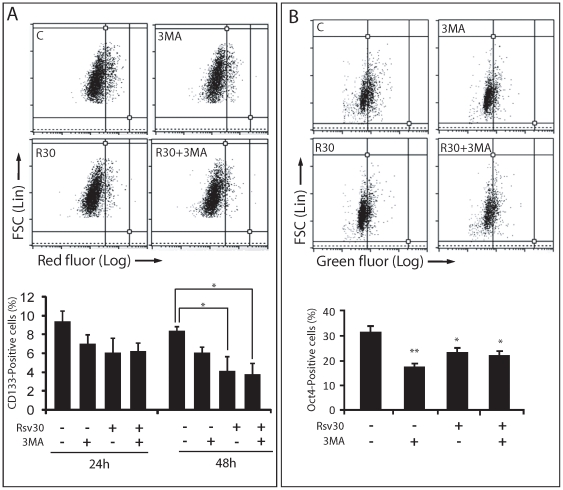
Rsv or autophagy inhibition reduces the proportion of CD133 and OCT4-positive cells in gliomas. **(A)** U87 cells were pre-incubated with 2 mM of 3MA for 1 h, treated with Rsv 30 µM for 24 or 48 h, followed by quantification of CD133 by flow cytometry; top plots – representative plots of each treatment. Graph indicates the percentage ± SEM of CD133 positive cells. **(B)** U87 cells were treated as in (A), and OCT4 was analyzed using a flow cytometry. Graph indicates the percentage ± SEM of OCT4 positive cells. *p>0.01 and *p>0.001 in relation to untreated cells.

Finally, because the increasingly importance described to CSCs in the resistance and maintenance of GBM, we tested the effect of Rsv and autophagy in the biology of these cells. We firstly tested the effect of our treatments on the formation of spheres, a typical feature of gCSCs and other types of CSCs [7]. Rsv at 30 µM, after 7 days of treatment, reduced the number of spheres to a very low level (10% of the number of spheres when compared to untreated cells) that precluded the evaluation of the role of autophagy in the formation of spheres *(data not show)*. Therefore, we chose the doses of 1 and 10 µM of Rsv, for a 7 day treatment. While Rsv 1 µM had no influence on sphere formation, Rsv 10 µM significantly reduced sphere formation (Fig. 5B). In the same way, inhibition of Rsv-induced autophagy, confirmed by AO staining (Fig. 5C) did not affect the reduction induced by Rsv 10 µM. Interestingly, inhibition of basal autophagy with 3MA reduced the number of spheres suggesting that basal autophagy helps to maintain the spherogenicity of gCSCs (Fig. 5A).Exploring the markers described for CSCs, we also evaluate the effect of Rsv in the percentage of CD133 and OCT4-positive cells. Corroborating our data from sphere formation assay, 3MA and Rsv 30 µM reduced the percentage of CD133-positive cells to around 70% and 33% of control, respectively, after 48 h. (Fig. 6A). The percentage of OCT4-positive cells was also reduced to around 50% and 70% of the control with 3MA or Rsv, respectively (Fig. 6B). Confirming the effect of the sphere formation assay, inhibition of Rsv-induced autophagy did not alter significantly the percentage of CD133 and OCT4-positive cells in relation to Rsv alone (Fig. 6A and B).

## Discussion

More than 30 molecular targets were described for Rsv, but several of these studies used Rsv in the middle or high micromolar range, were it can act on several targets and thus induces several kinds of processes [56]. For example, Rsv at 100 µM induces activation of caspase-3 and LDH release in U87 cells [33] and apoptosis and autophagy in U251 glioma cells, together with cell cycle arrest at the G1 phase and upregulation of Bax [57]. At 50 µM, Rsv induces release of cytochrome C from the mitochondria, formation of apoptosome, autophagic morphology and loss of membrane integrity in ovarian cancer cells [30], in a mechanism that was not inhibited by Bcl-2 overexpression, suggesting that neither classical apoptosis nor beclin1-dependent autophagy are involved. Since attainable concentrations in the circulation of rodents are in the low micromolar range [58], it is important to establish which of the several effects observed with Rsv (and other potential chemotherapeutic drugs) occur at the lower concentration range and whether these multiple effects are somehow linked.

Here we show that Rsv, at the relative low dose of 30 µM, inhibits the growth of glioma cells through a mechanism that involves autophagy, which modulates cell cycle arrest and apoptosis. Rsv induces autophagy, which plays a fundamental role in the S-G2/M arrest, since blockage of autophagy completely cancels out this effect. Rsv-induced autophagy has also a negative effect on apoptosis, since blocking autophagy unleashes apoptosis. However, if both apoptosis and autophagy are inhibited, the reduction in cell number induced by Rsv is reverted. Rsv alone induces a small increase in Bax and cleaved caspase-3 levels, suggesting that, although Rsv activates an apoptosis-inducing signal by itself, it is dominantly blocked by autophagy. The crosstalk between these three processes may be mitochondria-mediated [59], since damaged mitochondria are a common source of pro-apoptotic signals. According to this hypothesis, it is thought that removal of these organelles through mitophagy, the autophagy of mitochondria, avoids apoptotic cell death [60], while inhibition of mitophagy triggers it [61]. On the other hand, while mitophagy seems to be cell cycle-dependent [62], decrease of mitochondria delays cell cycle progression [63]. Thus, autophagy induced by Rsv may reduce cell number due to cell cycle arrest, but has a protective effect due to inhibition of apoptosis, which becomes clear when autophagy is inhibited. This may be mediated by targeting the mTor/Akt/p70S6K pathway which is inhibited by Rsv and is involved in control of mechanisms of cell growth and death, including cell cycle [64], apoptosis and autophagy [65].

It is thought that p53 plays a very important role in autophagy regulation [66], [67]. Here, Rsv-induced autophagosome formation was significantly lower in two p53 mutated cell lines, when compared to p53 wild type cells, suggesting that p53 may be part of, but not essential for, the mechanisms used by Rsv to induce autophagy. In accordance to this, *Geng et al* recently showed that chloroquine-induced autophagy is p53 independent on glioma cells such as U87 and U251 [68]. Moreover, p53 status may not be directly related to the sensitivity of glioma cells to Rsv, since the p53 negative cell lines present a similar or even higher decrease in cell number when compared to p53 proficient cells in our study.

Cell cycle analysis showed that Rsv induced a transient S-G2/M cell cycle arrest accompanied by inhibition of DNA synthesis after 24 h. This is suggestive of stalled DNA synthesis and a transient arrest in S phase. This is further supported by an increase in the levels of cyclin A and E, and pRb (S807/811). In agreement with this, *Rosenberg et al* demonstrated that overexpression of cyclin A causes premature entry into S-phase [69] and also induces a prolongation of S-phase and chromosomal double-strand break [70]. On the other hand, Rsv increased cyclin B and pCdc2(Y15) levels. In this case, the complex cyclin B/Cdc2 is formed but remains non-functional, since the critical step for its function as a kinase is the dephosphorylation on Tyr15 by the phosphatase Cdc25C [71]. A similar effect was observed by *Tyagi et al* in ovarian cancer cells [72], where Rsv also induced autophagy [30], but, in this case, the authors did not correlate autophagy and cell cycle arrest.

Inhibition of autophagy abrogates S-G2/M cell cycle arrest and partially reverts the block of DNA synthesis induced by Rsv, significantly reducing the levels of cyclin A, E, B and pRb(S807/811) and, mainly, pCdc(Y15). *Tasdemir et al* showed that formation of autophagic vacuoles occurs preferentially at G1 and, to a lesser extent, at G2, but none of the inducers of autophagy tested altered cell cycle distribution, at least in the HCT116 cell line [53]. Subsequently, it was shown by the same group, that p53 is part of the control of the relation between autophagy induction and cell cycle dynamic [53]. Moreover, in Hela cells, physiologic-like starvation reduces cyclin D and E levels, which was abrogated by inhibiting autophagy with 3MA [73], indicating a possible mechanism of interaction between autophagy and cell cycle regulation.

Inhibition of Rsv-induced autophagy triggered apoptosis in U87 cells, with an increase of Bax and cleaved caspase-3. Other glioma cells presented a similar phenotype, such as U373 treated with TMZ [45] and U251 treated with arsenic trioxide (As_2_O_3_) [74], and a considerable amount of work is being done on the mechanisms of this interaction [52]. Overexpression of Atg5 was shown to sensitize cells to apoptosis *in vitro* and *in vivo*, and this event was associated with cleavage of Atg5 by calpain, putting Atg5 in the core of the crosstalk between apoptosis and autophagy [75]. Recently, *Wirawan et al* showed that cleavage of beclin-1 by caspases inhibited autophagy and triggered apoptosis through association of C-terminal fragment of beclin-1 (a BH3-only protein of the Bcl-2 family) to mitochondria and liberation of proapoptotic factors, in a positive loop of apoptosis activation [76].

There is a growing body of evidence pointing to the importance of CSCs in the growth, survival and resistance of cancers to therapy. In glioma, there is good evidence for the selective resistance of gCSCs to γ-radiation [11], but for TMZ the two reports found point to different directions [77], [78]. Our data shows that 7 days treatment of Rsv at 10 µM reduces tumorsphere growth and, in 48 h with 30 µM, reduces the proportion of cells positive to markers of gCSCs, CD133 and OCT4, suggesting that Rsv induced either selective death or differentiation of gCSCs. Evidences in favor of a selective targeting of CSCs by Rsv were also obtained for breast cancer cells [79], medulloblastoma, in which Rsv led to radiosensitization [80] and pancreatic CSCs [81]. Chronic administration of Rsv 100 µM induced senescence and a glial- and neuronal-like differentiation of GBM cells, reducing its tumorigenicity [82]. It is also important to point out that chronic effects at low doses are especially important considering in vivo studies, where high micromolar concentration doses are very difficult to attain, especially in the central nervous system [83], [84]

The role of autophagy in the biology of CSCs has just started to be explored. We observed that inhibition of basal autophagy with 3MA reduces tumoresphere growth and the proportion of OCT4 positive cells, suggesting that basal autophagy is important for the survival or maintenance of the undifferentiated state of gCSCs. However, autophagy does not seem to be the key for the reduction in gCSCs induced by Rsv. Autophagy seems to act as a protective mechanism in the resistance to γ-radiation in gCSCs [85] since higher levels of autophagy are triggered in CD133+ cells treated with radiation when compared to CD133- cells [77].

The role of autophagy in cancer has been increasingly discussed. Monoallelic deletion of beclin 1 increased the incidence of tumor formation in mice, and the gene that encodes beclin 1 maps to a tumor susceptibility locus frequently deleted in breast, ovarian and prostate cancer [86], [87]. Moreover, cancer cells have an intrinsic capacity to evade apoptosis [38], [88], and this feature is widely present in GBM cells. Indeed, several works indicated that GBM cells seem to be more prone to therapies that induce autophagy rather than apoptosis [89], while cell cycle modulation is also a promising approach for the development of cancer therapies, including for GBM [38], [90]. Thus, the interactions among autophagy, apoptosis and cell cycle regulation, as well as a greater knowledge about the biology of gCSCs, are fundamental to understand tumoral biology and tailor therapeutic interventions not only to GBM, but also to other types of cancer.

## Materials and Methods

### Reagents

TMZ, Rsv, the inhibitor of phosphatidylinositol 3 kinase (PI3k) class III 3-methyladenine (3MA) and the fluorescent dye Acridine Orange (AO) were purchased from Sigma-Aldrich Chemical Co. (St. Louis, MO, USA). TMZ and Rsv were dissolved in dimethyl sulfoxide (DMSO, Acros Organics, NJ, USA). 3MA and AO were dissolved in water. Anti-Atg5, anti-Beclin1, anti-LC3, anti-phospho-Akt(Ser473), anti-Akt, anti-Bax, anti-cyclin D1, anti-cyclin A, anti-phospho Rb (S807/811), anti-OCT4, anti-rabbit Alexa-conjugate secondary antibody, anti-Pan-actin and anti-phospho-cdc2(Tyr15) were purchased from Cell Signaling Technology (Danvers, MA, USA). Anti-phospho-p70S6k, anti-cyclin B, anti-cyclin E and anti-caspase-3 were purchased from Santa Cruz Biotechnology, Inc (Santa Cruz, CA, USA). The pan caspase inhibitor zAsp-CH_2_-DCB (zAsp) (Peptide Institute, Tokyo, Japan), was kindly provided by Dr. Fabiana Horn (UFRGS) and dissolved in DMSO.

### Cell Culture and treatments

Human GBM cell lines U-87 MG, U-251 and U-138 MG (described only as U87, U251 and U138, respectively) were obtained from American Tissue Culture Collection (ATCC, Rockville, MD). All culture materials were purchased from Gibco Laboratories (Grand Island, NY, USA). Cells were cultured in DMEM low glucose supplemented with 10% fetal bovine serum (FBS), 1% penicillin/streptomycin and 0.1% amphoterecin B at 37°C and 5% CO_2_ in a humidified incubator. The inhibitors zAsp (100 µM) and/or 3MA (2 mM) were added 1 h before the treatments with Rsv. DMSO or water was added to the controls and did not exceed 0.5% (v/v).

### Cell counting and propidium iodide (PI) staining

Cells were counted in a hemocytometer and cell viability was analyzed by PI incorporation assay. To this end, U87 cells were seeded at 2×10^4^ cells per well in 24-well plates, treated as indicated, followed by cell counting. Number of cells is given as average ± SEM (standard error of de mean) in absolute number of cells or in relation to control, considered 100%, as indicated. For PI staining, treated cells were incubated with PI (6 µM) for 30 min and images were obtained using a Carl Zeiss Inverted Fluorescence Microscope (Carl Zeiss, Jena, Germany) with a rhodamine filter. The number of positively marked cells of at least 150 cells was determined in relation to the number of total cells, using the Image J software (NIH Image, Rockville, MD, USA).

### Analysis of autophagosome formation by LC3-GFP protein

Microtubule-associated protein 1 light chain 3 (MAP1-LC3 or only LC3) is a mammalian homologue of yeast Atg8p that translocates to the autophagosome membranes after lipidation [91]. U87 cells were transfected with the expression vector pEGFP-LC3, kindly provided by Dr. Tamotsu Yoshimori, using Lipofectamine 2000® according the manufacturer's instructions (Invitrogen, Carlsbad, CA, USA). Transfected cells were treated as indicated, followed by counting of at least 100 green cells per well [92], [93] and, among them, the percentage of cells that had at least 5 clear green dots in the cytosol was determined.

### Visualization and quantification of acidic vacuolar organelles (AVOs) by AO staining

AO is a marker of AVOs that fluoresces green in the whole cell except in acidic compartments (mainly late autophagosomes), where it fluoresces red. Development of AVOs is a typical feature of autophagy, and its formation indicates the maturation of autophagosomes and an efficient autophagic process, since only mature/late autophagosomes are acidic [31]. Cells were plated at 2×10^4^ cells per well in a 24-well plate, followed by treatments with Rsv and 3MA for the indicated times. After this, cells were incubated with AO (2.7 µM) for 15 min at room temperature, followed by visualization in a fluorescence microscope. Images were analyzed using Image J software. To quantify the percentage of cells with AVOs (red marked cells), treated cells were marked with AO, removed from the plate and analyzed by flow cytometry, as described previously [93] using a GUAVA flow cytometer and GUAVA Cytosoft (Millipore, Billerica, MA).

### Annexin-V staining

Apoptosis induction was quantified by Annexin V-FLUOS Apoptosis Kit (Roche, Germany) according to the manufacturer's instructions. Briefly, U87 cells were plated at 2×10^4^ cells per well in a 24-wells plate, followed by treatments as indicated. After this, cells were harvested and incubated with a solution containing 6 µM of PI and Annexin for 30 min, as indicated by the manufacturer, followed by flow cytometry. Cisplatin 16.6 µM was used for 24 h as a positive control of induction of apoptosis [94].

### Cell cycle analysis

For cell cycle analysis, cells were plated at 2×10^4^ cells per well in a 24-well plate, followed by treatments as indicated. After treatments, cells were harvested and fixed in cold ethanol 70% v/v in phosphate-buffered saline (PBS) for at least 2 h. Fixed cells were washed with PBS and marked with a solution containing PI 6 µM, Triton X-100 and RNAse for 30 min, in the dark, at room temperature. DNA content was analyzed through flow cytometer.

### BrdU Incorporation Assay

BrdU incorporation assay (BD Pharmingen, San Diego, CA) was used to evaluate the synthesis of DNA. To this end, U87 cells were seeded at 5×10^3^ cells per well in a 12-well plate, followed by treatments with 3MA and/or Rsv 30 µM for 24 h, as indicated. Cells were incubated with BrdU 10 µM for 3 h (from 21 to 24 h after treatment), followed by fixation in cold ethanol 70%. After this, cells were washed with PBS and incubated with HCl 2N for 30 min at room temperature. Thereafter, cells were washed twice with PBS and incubated with the anti-BrdU FITC-conjugated antibody, diluted 1∶20 in a PBS-T solution (1% bovine serum albumin (BSA) 1 mg/mL and 0.5% Tween 20), for 1 h, in the dark, under agitation. Finally, cells were washed once with PBS-T and incubated with the same solution used for cell cycle analysis, followed by flow cytometry.

### Western Blot Analysis

Analysis of protein expression and phosphorylation was performed as described previously with minor modifications [18]. Briefly, cells were lysed, proteins were quantified by Peterson's Method, with modifications [95] and the same amount of proteins (40 µg) was separated by SDS-PAGE, electroblotted onto a PVDF membrane (Amersham Pharmacia Biotech, Piscataway, NJ, USA) and stained with Coomassie Blue, which was used as loading control (see Fig. S3 and [96]). Membrane was blocked in 5% skimmed milk/TBST for one hour and probed with the primary antibody for Atg5 (1∶500), beclin-1 (1∶1000), LC3 (1∶500), phospho-Akt (pAkt Ser473) (1∶1000), Akt (1∶1000), Bax (1∶1000), cyclin D1 (1∶1000), cyclin A (1∶500), phospho-Rb (S807/811) (1∶1000), phospho-Cdc2 (Tyr15) (1∶1000), phospho-p70S6k (1∶1000), cyclin B (1∶250), cyclin E (1∶500) and caspase-3 (1∶1000) for 1 h at room temperature. Primary antibody was detected by incubating with appropriate horseradish peroxidase (HRP) conjugated-secondary antibody (1∶2000; Cell Signaling) for 2 h, using ECL and X-ray films (Kodak X-Omat, Rochester, NY, USA). Optical density of the bands was obtained using Bio-Rad software (Quantity One; Hercules, CA).

### Sphere Formation Assay

Glioblastoma CSCs have the capacity to form spheres *in vitro* and this “tumorsphere” formation reflect the stemness of the cell population [7], [97]. U87 cells were seeded at a density of 1×10^3^ cells per well in a 96-well plate. Cells were treated with 3MA and/or Rsv, as indicated, followed by counting the number of spheres formed in each condition. On day 4, medium was changed and treatments were re-added, and number of spheres was counted until day 7. In parallel, cells were tested for autophagy levels using AO, as cited above. After treatment, protein was quantified as to western blot analysis, to correct the number of spheres to the alterations in cell number induced by the treatments.

### CD133 and OCT4 Immunoassaying and Flow Cytometry Assays

CD133 (prominin-1) and OCT4 are markers of neural stem cells, including gCSCs [7], [98]. U87 cells were seeded at a density of 5×10^3^ cells per well in a 12-well plate. In order to analyze CD133, cells were mechanically dissociated and washed once with a PBS solution containing EDTA 2 mM and 0.5% of BSA for 1 h. After this, cells were incubated with PE-conjugated anti-CD133 (Miltenyi Biotec, Germany) 1∶10 (v/v in the solution cited above), for 10 min. After this, cells were analyzed by flow cytometry. For OCT4, cells were fixed with 4% formaldehyde (v/v in PBS) for 10 min at room temperature, followed by 1 min on ice. After this, cells were permeabilized with cold methanol 90% for at least 30 min on ice. Then, cells were centrifuged and resuspended in incubation buffer (0.5% BSA in PBS) for 10 min, followed by addition of primary antibody anti-OCT4, in a final dilution of 1∶200, for 3 h at room temperature. Thereafter, cells were washed with incubation buffer once, followed by incubation with secondary antibody conjugated to Alexa, diluted 1∶1000, for 30 min at room temperature in the dark. Finally, cells were washed once with incubation buffer, resuspended and analyzed by flow cytometry.

### Statistical Analysis

All experiments were done at least three times independently and in triplicate. Statistical analysis was conducted by ANOVA followed by SNK (Student Newmans Keuls) post-hoc test to multiple comparisons. ‘*p*’ value under 0.05 was considered significant.

## Supporting Information

Figure S1 **Inhibition of autophagy induced by Rsv leads to apoptotic phenotype.** Morphology of cells treated with Rsv (30 µM) in the presence or not of 3MA.
(TIF)

Figure S2 **Resveratrol induces S-G2/M cell cycle arrest in U251 and U138 cells.** **(A)** U251 and U138 cells were treated with Rsv 30 µM for 48 h and cell cycle was analyzed by flow cytometry. Numbers represent the average of the percentage of cells in each phase; * p<0.05, *** p<0.01;
(TIF)

Figure S3 **Comparison of the intensities of actin and coomassie blue stained membrane used as loading controls.**
(TIF)

## References

[1] Maher EA, Furnari FB, Bachoo RM, Rowitch DH, Louis DN (2001). Malignant glioma: genetics and biology of a grave matter.. Genes Dev.

[2] Furnari FB, Fenton T, Bachoo RM, Mukasa A, Stommel JM (2007). Malignant astrocytic glioma: genetics, biology, and paths to treatment.. Genes Dev.

[3] Ohgaki H, Kleihues P (2007). Genetic pathways to primary and secondary glioblastoma.. Am J Pathol.

[4] Ricci-Vitiani L, Pallini R, Biffoni M, Todaro M, Invernici G (2010). Tumour vascularization via endothelial differentiation of glioblastoma stem-like cells.. Nature.

[5] Wang R, Chadalavada K, Wilshire J, Kowalik U, Hovinga KE (2010). Glioblastoma stem-like cells give rise to tumour endothelium.. Nature.

[6] Lenz G (2010). Transient oncogenes.. Med Hypotheses.

[7] Singh SK, Clarke ID, Terasaki M, Bonn VE, Hawkins C (2003). Identification of a cancer stem cell in human brain tumors.. Cancer Res.

[8] Wang R, Chadalavada K, Wilshire J, Kowalik U, Hovinga KE (2010). Glioblastoma stem-like cells give rise to tumour endothelium.. Nature.

[9] Fan QW, Cheng C, Hackett C, Feldman M, Houseman BT (2010). Akt and autophagy cooperate to promote survival of drug-resistant glioma.. Sci Signal.

[10] Huang Q, Zhang QB, Dong J, Wu YY, Shen YT (2008). Glioma stem cells are more aggressive in recurrent tumors with malignant progression than in the primary tumor, and both can be maintained long-term in vitro.. BMC Cancer.

[11] Bao S, Wu Q, McLendon RE, Hao Y, Shi Q (2006). Glioma stem cells promote radioresistance by preferential activation of the DNA damage response.. Nature.

[12] Aoki T, Hashimoto N, Matsutani M (2007). Management of glioblastoma.. Expert Opin Pharmacother.

[13] Mason WP, Cairncross JG (2005). Drug Insight: temozolomide as a treatment for malignant glioma–impact of a recent trial.. Nat Clin Pract Neurol.

[14] Stupp R, Mason WP, van den Bent MJ, Weller M, Fisher B (2005). Radiotherapy plus concomitant and adjuvant temozolomide for glioblastoma.. N Engl J Med.

[15] Lagouge M, Argmann C, Gerhart-Hines Z, Meziane H, Lerin C (2006). Resveratrol improves mitochondrial function and protects against metabolic disease by activating SIRT1 and PGC-1alpha.. Cell.

[16] Shakibaei M, Harikumar KB, Aggarwal BB (2009). Resveratrol addiction: to die or not to die.. Mol Nutr Food Res.

[17] Dasgupta B, Milbrandt J (2007). Resveratrol stimulates AMP kinase activity in neurons.. Proc Natl Acad Sci U S A.

[18] Zamin LL, Dillenburg-Pilla P, Argenta-Comiran R, Horn AP, Simao F (2006). Protective effect of resveratrol against oxygen-glucose deprivation in organotypic hippocampal slice cultures: Involvement of PI3-K pathway.. Neurobiol Dis.

[19] Jang M, Cai L, Udeani GO, Slowing KV, Thomas CF (1997). Cancer chemopreventive activity of resveratrol, a natural product derived from grapes.. Science.

[20] Pozo-Guisado E, Merino JM, Mulero-Navarro S, Lorenzo-Benayas MJ, Centeno F (2005). Resveratrol-induced apoptosis in MCF-7 human breast cancer cells involves a caspase-independent mechanism with downregulation of Bcl-2 and NF-kappaB.. Int J Cancer.

[21] Delmas D, Rebe C, Lacour S, Filomenko R, Athias A (2003). Resveratrol-induced apoptosis is associated with Fas redistribution in the rafts and the formation of a death-inducing signaling complex in colon cancer cells.. J Biol Chem.

[22] Fuggetta MP, D'Atri S, Lanzilli G, Tricarico M, Cannavo E (2004). In vitro antitumour activity of resveratrol in human melanoma cells sensitive or resistant to temozolomide.. Melanoma Res.

[23] Sexton E, Van Themsche C, LeBlanc K, Parent S, Lemoine P (2006). Resveratrol interferes with AKT activity and triggers apoptosis in human uterine cancer cells.. Mol Cancer.

[24] Whyte L, Huang YY, Torres K, Mehta RG (2007). Molecular mechanisms of resveratrol action in lung cancer cells using dual protein and microarray analyses.. Cancer Res.

[25] Estrov Z, Shishodia S, Faderl S, Harris D, Van Q (2003). Resveratrol blocks interleukin-1beta-induced activation of the nuclear transcription factor NF-kappaB, inhibits proliferation, causes S-phase arrest, and induces apoptosis of acute myeloid leukemia cells.. Blood.

[26] Joe AK, Liu H, Suzui M, Vural ME, Xiao D (2002). Resveratrol induces growth inhibition, S-phase arrest, apoptosis, and changes in biomarker expression in several human cancer cell lines.. Clin Cancer Res.

[27] Mahyar-Roemer M, Katsen A, Mestres P, Roemer K (2001). Resveratrol induces colon tumor cell apoptosis independently of p53 and precede by epithelial differentiation, mitochondrial proliferation and membrane potential collapse.. Int J Cancer.

[28] Scifo C, Cardile V, Russo A, Consoli R, Vancheri C (2004). Resveratrol and propolis as necrosis or apoptosis inducers in human prostate carcinoma cells.. Oncol Res.

[29] Bhardwaj A, Sethi G, Vadhan-Raj S, Bueso-Ramos C, Takada Y (2007). Resveratrol inhibits proliferation, induces apoptosis, and overcomes chemoresistance through down-regulation of STAT3 and nuclear factor-kappaB-regulated antiapoptotic and cell survival gene products in human multiple myeloma cells.. Blood.

[30] Opipari AW, Tan L, Boitano AE, Sorenson DR, Aurora A (2004). Resveratrol-induced autophagocytosis in ovarian cancer cells.. Cancer Res.

[31] Klionsky DJ, Abeliovich H, Agostinis P, Agrawal DK, Aliev G (2008). Guidelines for the use and interpretation of assays for monitoring autophagy in higher eukaryotes.. Autophagy.

[32] Zamin LL, Filippi-Chiela EC, Dillenburg-Pilla P, Horn F, Salbego C (2009). Resveratrol and quercetin cooperate to induce senescence-like growth arrest in C6 rat glioma cells.. Cancer Sci.

[33] Jiang H, Zhang L, Kuo J, Kuo K, Gautam SC (2005). Resveratrol-induced apoptotic death in human U251 glioma cells.. Mol Cancer Ther.

[34] Li J, Qin Z, Liang Z (2009). The prosurvival role of autophagy in Resveratrol-induced cytotoxicity in human U251 glioma cells.. BMC Cancer.

[35] Lefranc F, Brotchi J, Kiss R (2005). Possible future issues in the treatment of glioblastomas: special emphasis on cell migration and the resistance of migrating glioblastoma cells to apoptosis.. J Clin Oncol.

[36] Green DR (2005). Apoptotic pathways: ten minutes to dead.. Cell.

[37] Kroemer G, Jaattela M (2005). Lysosomes and autophagy in cell death control.. Nat Rev Cancer.

[38] Okada H, Mak TW (2004). Pathways of apoptotic and non-apoptotic death in tumour cells.. Nat Rev Cancer.

[39] Lefranc F, Kiss R (2008). The sodium pump alpha1 subunit as a potential target to combat apoptosis-resistant glioblastomas.. Neoplasia.

[40] Baehrecke EH (2005). Autophagy: dual roles in life and death?. Nat Rev Mol Cell Biol.

[41] Shintani T, Mizushima N, Ogawa Y, Matsuura A, Noda T (1999). Apg10p, a novel protein-conjugating enzyme essential for autophagy in yeast.. Embo J.

[42] Levine B, Yuan J (2005). Autophagy in cell death: an innocent convict?. J Clin Invest.

[43] Miracco C, Cosci E, Oliveri G, Luzi P, Pacenti L (2007). Protein and mRNA expression of autophagy gene Beclin 1 in human brain tumours.. Int J Oncol.

[44] Chen Q, Xie W, Kuhn DJ, Voorhees PM, Lopez-Girona A (2008). Targeting the p27 E3 ligase SCF(Skp2) results in p27- and Skp2-mediated cell-cycle arrest and activation of autophagy.. Blood.

[45] Kanzawa T, Germano IM, Komata T, Ito H, Kondo Y (2004). Role of autophagy in temozolomide-induced cytotoxicity for malignant glioma cells.. Cell Death Differ.

[46] Lefranc F, Kiss R (2006). Autophagy, the Trojan horse to combat glioblastomas.. Neurosurg Focus.

[47] Ito H, Daido S, Kanzawa T, Kondo S, Kondo Y (2005). Radiation-induced autophagy is associated with LC3 and its inhibition sensitizes malignant glioma cells.. Int J Oncol.

[48] Kanzawa T, Zhang L, Xiao L, Germano IM, Kondo Y (2005). Arsenic trioxide induces autophagic cell death in malignant glioma cells by upregulation of mitochondrial cell death protein BNIP3.. Oncogene.

[49] Eshleman JS, Carlson BL, Mladek AC, Kastner BD, Shide KL (2002). Inhibition of the mammalian target of rapamycin sensitizes U87 xenografts to fractionated radiation therapy.. Cancer Res.

[50] Lefranc F, Facchini V, Kiss R (2007). Proautophagic drugs: a novel means to combat apoptosis-resistant cancers, with a special emphasis on glioblastomas.. Oncologist.

[51] Alva AS, Gultekin SH, Baehrecke EH (2004). Autophagy in human tumors: cell survival or death?. Cell Death Differ.

[52] Maiuri MC, Zalckvar E, Kimchi A, Kroemer G (2007). Self-eating and self-killing: crosstalk between autophagy and apoptosis.. Nat Rev Mol Cell Biol.

[53] Vicencio JM, Galluzzi L, Tajeddine N, Ortiz C, Criollo A (2008). Senescence, apoptosis or autophagy? When a damaged cell must decide its path–a mini-review.. Gerontology.

[54] Seglen PO, Gordon PB (1982). 3-Methyladenine: specific inhibitor of autophagic/lysosomal protein degradation in isolated rat hepatocytes.. Proc Natl Acad Sci U S A.

[55] Harikumar KB, Aggarwal BB (2008). Resveratrol: a multitargeted agent for age-associated chronic diseases.. Cell Cycle.

[56] Pirola L, Frojdo S (2008). Resveratrol: one molecule, many targets.. IUBMB Life.

[57] Gu J, Liu Y, Kyritsis AP, Bondy ML (2009). Molecular epidemiology of primary brain tumors.. Neurotherapeutics.

[58] Asensi M, Medina I, Ortega A, Carretero J, Bano MC (2002). Inhibition of cancer growth by resveratrol is related to its low bioavailability.. Free Radic Biol Med.

[59] Lemasters JJ, Nieminen AL, Qian T, Trost LC, Elmore SP (1998). The mitochondrial permeability transition in cell death: a common mechanism in necrosis, apoptosis and autophagy.. Biochim Biophys Acta.

[60] Elmore SP, Qian T, Grissom SF, Lemasters JJ (2001). The mitochondrial permeability transition initiates autophagy in rat hepatocytes.. FASEB J.

[61] Boya P, Gonzalez-Polo RA, Casares N, Perfettini JL, Dessen P (2005). Inhibition of macroautophagy triggers apoptosis.. Mol Cell Biol.

[62] Tasdemir E, Maiuri MC, Tajeddine N, Vitale I, Criollo A (2007). Cell cycle-dependent induction of autophagy, mitophagy and reticulophagy.. Cell Cycle.

[63] Byun HO, Kim HY, Lim JJ, Seo YH, Yoon G (2008). Mitochondrial dysfunction by complex II inhibition delays overall cell cycle progression via reactive oxygen species production.. J Cell Biochem.

[64] Sun H, Lesche R, Li DM, Liliental J, Zhang H (1999). PTEN modulates cell cycle progression and cell survival by regulating phosphatidylinositol 3,4,5,-trisphosphate and Akt/protein kinase B signaling pathway.. Proc Natl Acad Sci U S A.

[65] Vivanco I, Sawyers CL (2002). The phosphatidylinositol 3-Kinase AKT pathway in human cancer.. Nat Rev Cancer.

[66] Crighton D, Wilkinson S, Ryan KM (2007). DRAM links autophagy to p53 and programmed cell death.. Autophagy.

[67] Jin S (2005). p53, Autophagy and tumor suppression.. Autophagy.

[68] Geng Y, Kohli L, Klocke B, Roth K (2010). Chroloquine-induced autophagic vacuole accumulation and cell death in glioma cells is p53 independent. Neuro-Oncol.

[69] Rosenberg AR, Zindy F, Le Deist F, Mouly H, Metezeau P (1995). Overexpression of human cyclin A advances entry into S phase.. Oncogene.

[70] Tane S, Chibazakura T (2009). Cyclin A overexpression induces chromosomal double-strand breaks in mammalian cells.. Cell Cycle.

[71] Hoffmann I, Karsenti E (1994). The role of cdc25 in checkpoints and feedback controls in the eukaryotic cell cycle.. J Cell Sci.

[72] Tyagi A, Singh RP, Agarwal C, Siriwardana S, Sclafani RA (2005). Resveratrol causes Cdc2-tyr15 phosphorylation via ATM/ATR-Chk1/2-Cdc25C pathway as a central mechanism for S phase arrest in human ovarian carcinoma Ovcar-3 cells.. Carcinogenesis.

[73] Tasdemir E, Galluzzi L, Maiuri MC, Criollo A, Vitale I (2008). Methods for assessing autophagy and autophagic cell death.. Methods Mol Biol.

[74] Kanzawa T, Kondo Y, Ito H, Kondo S, Germano I (2003). Induction of autophagic cell death in malignant glioma cells by arsenic trioxide.. Cancer Res.

[75] Yousefi S, Perozzo R, Schmid I, Ziemiecki A, Schaffner T (2006). Calpain-mediated cleavage of Atg5 switches autophagy to apoptosis.. Nat Cell Biol.

[76] Wirawan EVW, Vande Walle L, Kersse K, Cornelis S, Claerhout S (2010). Caspase-mediated cleavage of Beclin-1 inactivates Beclin-1-induced autophagy and enhances apoptosis by promoting the release of proapoptotic factors from mitochondria.. Cell Death and Disease.

[77] Fu J, Liu ZG, Liu XM, Chen FR, Shi HL (2009). Glioblastoma stem cells resistant to temozolomide-induced autophagy.. Chin Med J (Engl).

[78] Beier D, Rohrl S, Pillai DR, Schwarz S, Kunz-Schughart LA (2008). Temozolomide preferentially depletes cancer stem cells in glioblastoma.. Cancer Res.

[79] Pandey PR, Okuda H, Watabe M, Pai SK, Liu W (2010). Resveratrol suppresses growth of cancer stem-like cells by inhibiting fatty acid synthase.. Breast Cancer Res Treat.

[80] Lu KH, Chen YW, Tsai PH, Tsai ML, Lee YY (2009). Evaluation of radiotherapy effect in resveratrol-treated medulloblastoma cancer stem-like cells.. Childs Nerv Syst.

[81] Shankar S, Nall D, Tang SN, Meeker D, Passarini J (2011). Resveratrol inhibits pancreatic cancer stem cell characteristics in human and KrasG12D transgenic mice by inhibiting pluripotency maintaining factors and epithelial-mesenchymal transition.. PLoS One.

[82] Castino R, Pucer A, Veneroni R, Morani F, Peracchio C (2011). Resveratrol Reduces the Invasive Growth and Promotes the Acquisition of a Long-Lasting Differentiated Phenotype in Human Glioblastoma Cells.. J Agric Food Chem.

[83] Boocock DJ, Faust GE, Patel KR, Schinas AM, Brown VA (2007). Phase I dose escalation pharmacokinetic study in healthy volunteers of resveratrol, a potential cancer chemopreventive agent.. Cancer Epidemiol Biomarkers Prev.

[84] Sale S, Verschoyle RD, Boocock D, Jones DJ, Wilsher N (2004). Pharmacokinetics in mice and growth-inhibitory properties of the putative cancer chemopreventive agent resveratrol and the synthetic analogue trans 3,4,5,4′-tetramethoxystilbene.. Br J Cancer.

[85] Lomonaco SL, Finniss S, Xiang C, Decarvalho A, Umansky F (2009). The induction of autophagy by gamma-radiation contributes to the radioresistance of glioma stem cells.. Int J Cancer.

[86] Qu X, Yu J, Bhagat G, Furuya N, Hibshoosh H (2003). Promotion of tumorigenesis by heterozygous disruption of the beclin 1 autophagy gene.. J Clin Invest.

[87] Yue Z, Jin S, Yang C, Levine AJ, Heintz N (2003). Beclin 1, an autophagy gene essential for early embryonic development, is a haploinsufficient tumor suppressor.. Proc Natl Acad Sci U S A.

[88] Hanahan D, Weinberg RA (2000). The hallmarks of cancer.. Cell.

[89] Ziegler DS, Kung AL, Kieran MW (2008). Anti-apoptosis mechanisms in malignant gliomas.. J Clin Oncol.

[90] Collins K, Jacks T, Pavletich NP (1997). The cell cycle and cancer.. Proc Natl Acad Sci U S A.

[91] Kabeya Y, Mizushima N, Ueno T, Yamamoto A, Kirisako T (2000). LC3, a mammalian homologue of yeast Apg8p, is localized in autophagosome membranes after processing.. Embo J.

[92] Pattingre S, Tassa A, Qu X, Garuti R, Liang XH (2005). Bcl-2 antiapoptotic proteins inhibit Beclin 1-dependent autophagy.. Cell.

[93] Jiang H, White EJ, Conrad C, Gomez-Manzano C, Fueyo J (2009). Autophagy pathways in glioblastoma.. Methods Enzymol.

[94] Kondo S, Barna BP, Morimura T, Takeuchi J, Yuan J (1995). Interleukin-1 beta-converting enzyme mediates cisplatin-induced apoptosis in malignant glioma cells.. Cancer Res.

[95] Peterson GL (1983). Determination of total protein.. Methods Enzymol.

[96] Welinder C, Ekblad L (2011). Coomassie staining as loading control in Western blot analysis.. J Proteome Res.

[97] Yuan X, Curtin J, Xiong Y, Liu G, Waschsmann-Hogiu S (2004). Isolation of cancer stem cells from adult glioblastoma multiforme.. Oncogene.

[98] Kim JB, Sebastiano V, Wu G, Arauzo-Bravo MJ, Sasse P (2009). Oct4-induced pluripotency in adult neural stem cells.. Cell.

