# Supplementary figures and images for: Autophagy Interplay with Apoptosis and Cell Cycle Regulation in the Growth Inhibiting Effect of Resveratrol in Glioma Cells
Source: PLoS One. 2011 Jun 13;6(6):e20849. doi: 10.1371/journal.pone.0020849 (PMC3113895; doi:10.1371/journal.pone.0020849)

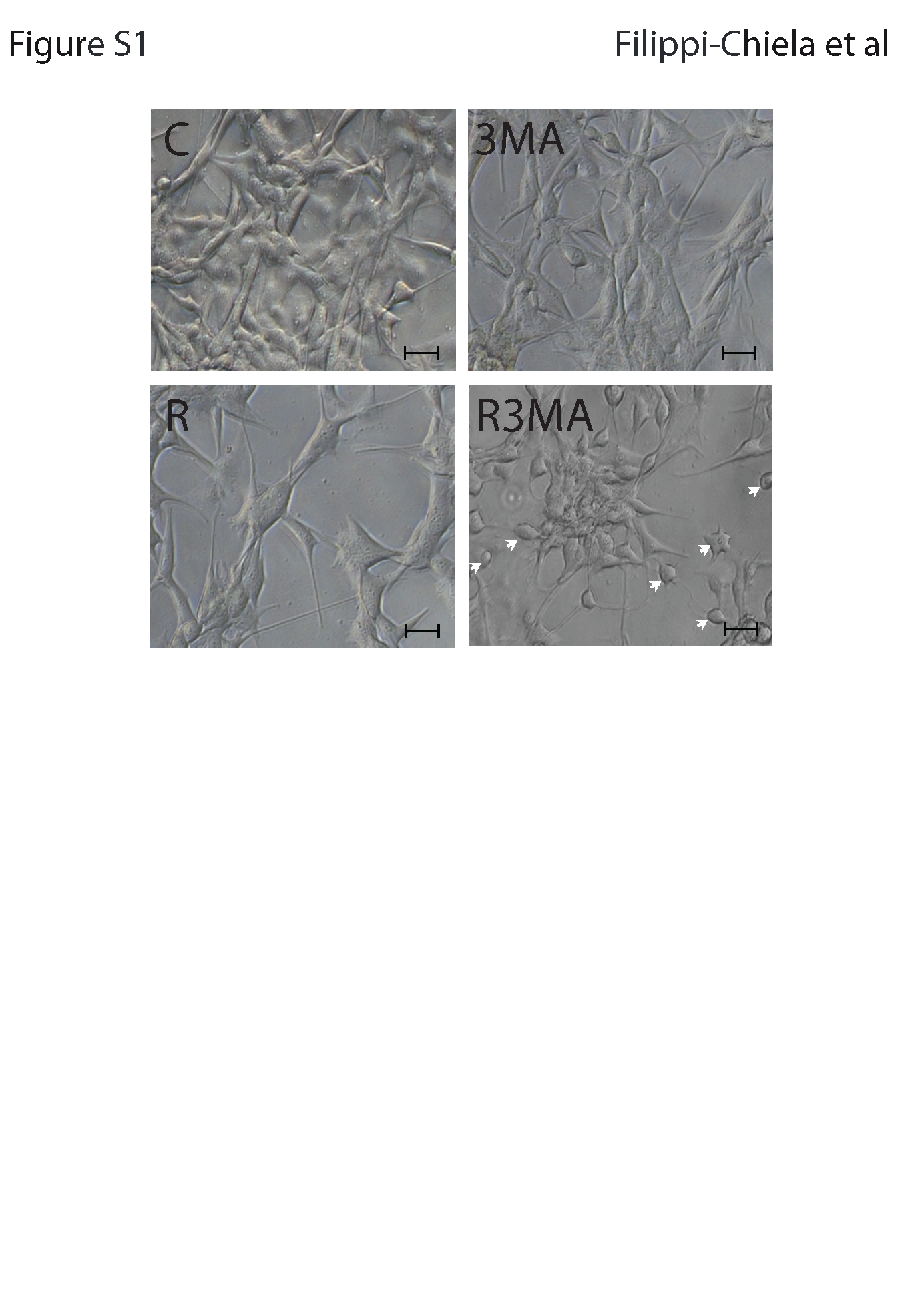

Supplement: Figure S1 — Inhibition of autophagy induced by Rsv leads to apoptotic phenotype. Morphology of cells treated with Rsv (30 µM) in the presence or not of 3MA. (TIF) [file pone.0020849.s001.tif]

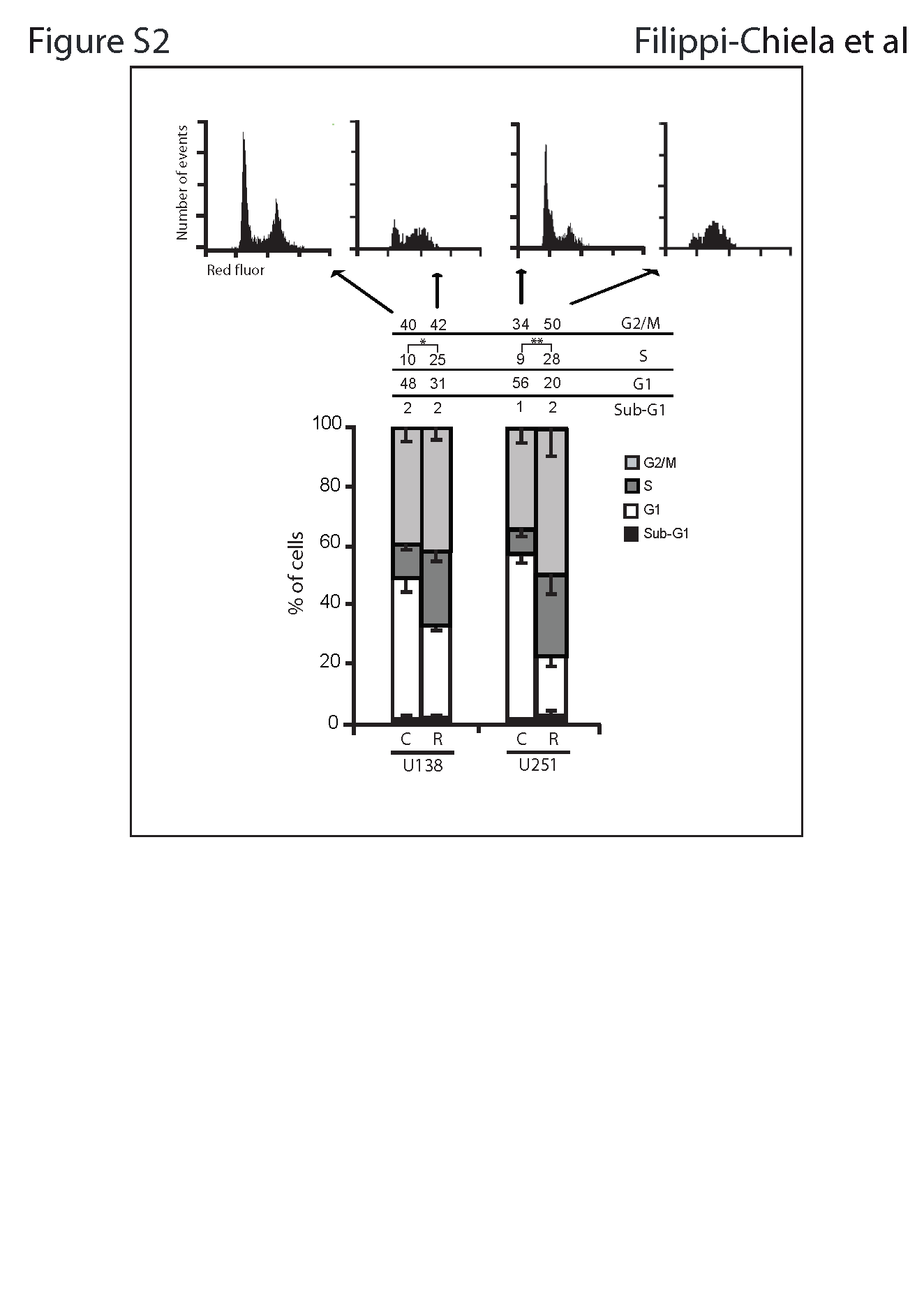

Supplement: Figure S2 — Resveratrol induces S-G2/M cell cycle arrest in U251 and U138 cells. (A) U251 and U138 cells were treated with Rsv 30 µM for 48 h and cell cycle was analyzed by flow cytometry. Numbers represent the average of the percentage of cells in each phase; * p<0.05, *** p<0.01; (TIF) [file pone.0020849.s002.tif]

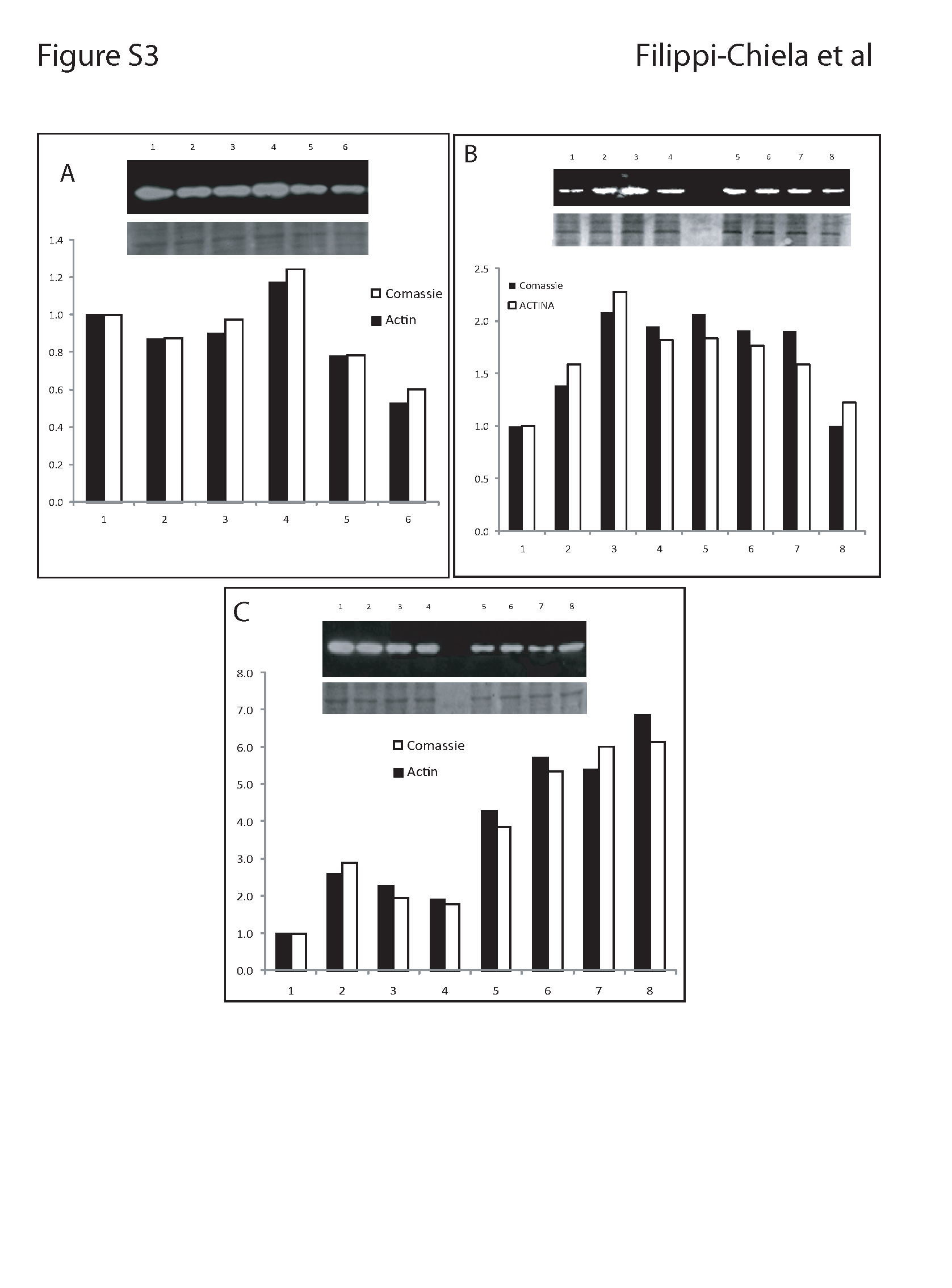

Supplement: Figure S3 — Comparison of the intensities of actin and coomassie blue stained membrane used as loading controls. (TIF) [file pone.0020849.s003.tif]
